# Hydrology‐driven responses of herbivorous geese in relation to changes in food quantity and quality

**DOI:** 10.1002/ece3.6272

**Published:** 2020-04-20

**Authors:** Pingyang Zhang, Ye‐ai Zou, Yonghong Xie, Siqi Zhang, Xinsheng Chen, Feng Li, Zhengmiao Deng, Hong Zhang, Wei Tu

**Affiliations:** ^1^ Key Laboratory of Agro‐ecological Processes in Subtropical Regions Chinese Academy of Sciences Changsha China; ^2^ Dongting Lake Station for Wetland Ecosystem Research Institute of Subtropical Agriculture Chinese Academy of Sciences Changsha China; ^3^ University of Chinese Academy of Sciences Beijing China; ^4^ Administrative Bureau of Hunan East Dongting Lake National Nature Reserve Yueyang China

**Keywords:** Bean goose, diet, Dongting Lake wetland, food shortage, habitat selection, Lesser white‐fronted goose

## Abstract

East Dongting Lake is a Ramsar site and a particularly important wintering ground for herbivorous geese along the East Asian‐Australasian Flyway. The operation of the Three Gorges Dam has changed the water regime and has a significant impact on wetland ecosystems downstream. We studied the responses of two sympatric herbivorous goose species, the Lesser white‐fronted goose *Anser erythropus* and Bean goose *Anser fabalis*, to habitat change by investigating their food conditions, habitat selection, and diet composition in the wintering periods of 2016/2017 and 2017/2018, which had early and late water recession, respectively. It was expected that the contrasting water regimes would result in different food conditions and geese responses. The results showed that the food quality and quantity differed significantly between winters. As responses to the high‐quantity/low‐quality food during 2016/2017, more geese switched to feeding on mudflat and exploited plants such as dicotyledons and moss. The tall swards of *Carex* spp. (dominant plants in the meadow) that developed during the first growing season decreased the food accessibility during the second growing season and hindered the exploitation of newly generated shoots by the geese, which was further confirmed by our clipping control experiment. Nearly all the geese chose to feed on meadow, and *Carex* spp. made up the majority of their diet in 2017/2018 when there was more low‐quantity/high‐quality food. Compared with the globally vulnerable Lesser white‐fronted geese, the larger‐sized Bean geese seemed to be less susceptible to winter food shortages and exhibited more stable responses. We concluded that the food quality–quantity condition was the external factor influencing the geese responses, while morphological and physiological traits could be the internal factors causing different responses between the two species. This study enhanced the understanding of the influence that habitat change exerts on herbivorous geese in their wintering site in the context of the Three Gorges Dam operation. We suggested that regulating hydrological regime was important in terms of wetland management and species conservation.

## Graphical abstract

1. Contrasting water regime in two successive wintering periods influenced by the Three Gorges Dam operation led to significant differences in food conditions and response of two dominant herbivorous goose species, Lesser white‐fronted goose Anser erythropus, and Bean goose Anser fabalis.
2. More geese, especially the Lesser white‐fronted geese, fed on mudflat as response to high‐quantity/low‐quality food caused by early water level recession, while nearly all the geese chose meadow and fed on the most abundance Carex spp. in the other wintering period when there were more low‐quantity/high‐quality food caused by late water recession.
3. The larger‐sized Bean geese were less susceptible to winter food shortages and exhibited more stable responses than Lesser white‐fronted geese, which were more vulnerable.

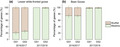

## 1 INTRODUCTION

Understanding how and to what extent organisms perceive and respond to spatiotemporal variability in their environment is a core feature of contemporary ecology. Waterbirds rely highly on wetlands, and their habitat and food conditions are largely driven by hydrological regimes. Water level and food availability are considered the major abiotic and biotic factors, respectively, that determine waterbirds occurrence (Hagy & Kaminski, 2012; Li, Yang, et al., 2019; Zou et al., 2019). More specifically, food quality and quantity may determine directly the body condition of waterbirds, while water level fluctuations may influence the abundance of and accessibility to food resources (Gawlik & Crozier, 2007; Royan, Hannah, Reynolds, Noble, & Sadler, 2013). Therefore, coping with challenges associated with food shortage is a key problem for waterbirds when trying to survive, especially in light of the recent global habitat loss and degradation (Fischer & Lindenmayer, 2007).

In the Yangtze River floodplain in China, the extensive and numerous ephemeral wetlands formed annually by flood recession provide critical wintering habitat and food resources for Anatidae species, especially herbivorous goose species (Jia et al., 2016). However, the flow control by the Three Gorges Dam has greatly influenced the water levels and eco‐hydrological environment of the lake wetlands downstream (Lai, Liang, Jiang, & Huang, 2014); one such example is the East Dongting Lake wetland, which is a Ramsar site along the East Asian‐Australasian Flyway. Normally, as water level recedes in autumn, large areas of exposed substrate support sufficient growth of meadow vegetation, which consists mainly of sedge *Carex* spp. communities (dominated by *Carex brevicuspis*; Chen, Xie, Deng, Li, & Hou, 2011). As herbivorous geese generally prefer short, better quality swards, the relationship among water level recession, wetland vegetation growth, and wintering geese arrival is important for geese survival during the wintering period. According to historical data and previous studies, overwintering geese generally arrive at East Dongting Lake in late October to early November, and the timing of optimal water drawdown should be early to mid‐October to ensure quality food sources for the geese (Guan, Lei, et al., 2016; Zou, Tang, Xie, Zhao, & Zhang, 2017). However, since the Three Gorges Dam started operating in 2003, the monthly water level from July to November decreased considerably and the submergence duration of grassland decreased obviously in Dongting Lake (Hu, Xie, Tang, Li, & Zou, 2018; Xie, Tang, Chen, Li, & Deng, 2015). As a result, herbivorous geese have been subjected to unfavorable habitat change and have been led to mistime their arrival during early winter (Zhao, Cong, Barter, Fox, & Cao, 2012).

The “forage maturation hypothesis” states that foragers optimize their fitness not only in terms of energy, but also by responding simultaneously to changes in food quantity and quality because they depend on a wide range of nutrients and the digestibility of prey items to meet their dietary requirements (Hebblewhite, Merrill, & McDermid, 2008; Raubenheimer, Simpson, & Mayntz, 2009). Plant nitrogen is a key nutrient for herbivores, and low fiber content is more favorable as it may increase the food digestibility and palatability (Berner, Blanckenhorn, & Körner, 2005; Veloso & Bozinovic, 1993). Plant quality generally decreases with growing season; as sward height increases, nitrogen content decreases and fiber content increases (Stejskalová, Hejcmanová, Pavlů, & Hejcman, 2013; Van Der Wal et al., 2000). Consumers’ behavior is usually affected by food quantity–quality changes (Burian, Nielsen, & Winder, 2019). We then expected that, in East Dongting Lake, the wintering geese will respond to changes in food quantity and quality accordingly in aspects of habitat and diet selection, so as to maximize their fitness. On the other hand, interspecific differences often occur when species are facing the same habitat change. Durant, Fritz, and Duncan (2004) underlined the role of body size as an important cause of variations in the grazing patterns of herbivores. The functional response of herbivores, which describes how the instantaneous intake rate changes with increasing food availability, is highly associated with body size (Durant, Fritz, Blais, & Duncan, 2003). Different‐sized herbivores may respond differently to sward height changes in terms of their in intake rate and patch selection (Zhang et al., 2016).

The target species in this study are Lesser white‐fronted goose *Anser erythropus* and Bean goose *Anser fabalis*. They differ significantly in terms of body size (Lesser white‐fronted geese 53–66 cm; Bean geese 80–90 cm). We expected that the two sympatric species in East Dongting Lake would exhibit different response when facing the same variations in food supply. East Dongting Lake supports nearly 90% of the Eastern Palearctic wintering population of Lesser white‐fronted goose (Wang, Fox, Cong, Barter, & Cao, 2012), which is listed as vulnerable on the IUCN Red List, with its global population estimate still decreasing (IUCN, 2020). The population wintering in Dongting Lake breeds in southern Taimyr and areas north of Eastern Siberia and Chukotka, and possibly stopover in Mongolia (IUCN, 2020). Bean goose is one of the most abundant waterbird species wintering in Dongting Lake. This population may breed in northeast Siberia and tends to stopover at the Northeast China Plain (Si et al., 2018). In East Dongting wetland, the two goose species largely feed on recessional sedge meadows and short grassland and exploit similar food resources, such as *Carex*, *Alopecurus*, *Polygonum,* and *Phalaris* spp. (Fox et al., 2008; Guan, Debashish, et al., 2016). We considered that the responses of these two goose species to habitat and food changes caused by varied water regime are an important issue in terms of habitat management and biodiversity conservation.

Despite the importance of the two goose species in East Dongting Lake wetland, studies on their feeding ecology under the influence of the Three Gorges Dam project are scarce. In this study, the water‐level fluctuations between two successive wintering periods (2016/2017 and 2017/2018) were contrasting owing to the manipulation of the Three Gorges Dam. We measured the variations in both food quantity and quality as cues explaining the responses of the two herbivorous goose species in terms of habitat selection and diet composition. A clipping control experiment was included for further explanation. We aimed to explore: (a) how the food conditions were affected by the altered water regime, (b) how the geese responded regarding changes in food quality and quantity, and (c) whether the two herbivorous goose species responded differently as expected.

## 2 MATERIALS AND METHODS

### 2.1 Study site

**FIGURE 1:**
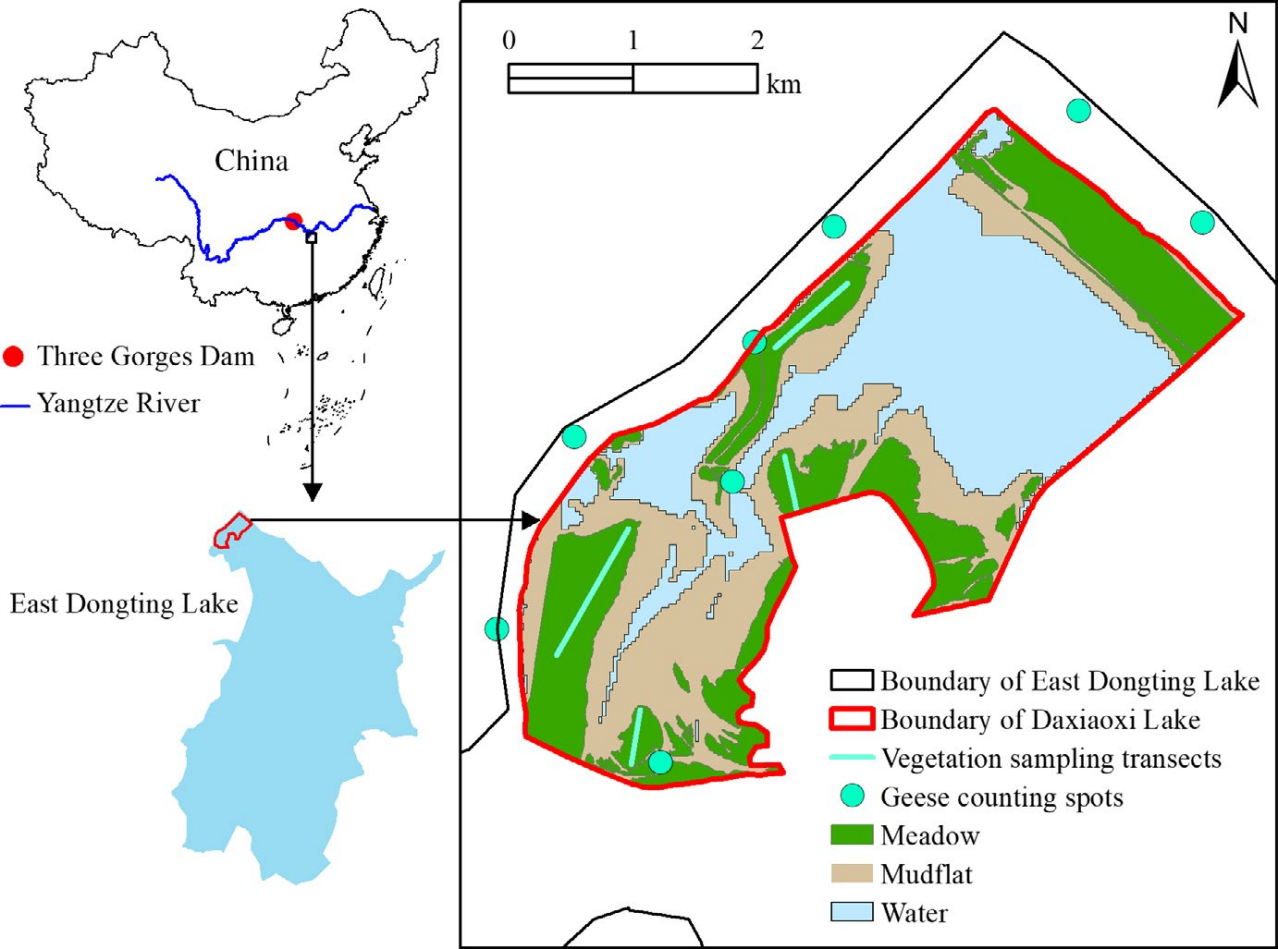
Geographical location of East Dongting Lake and the sampling sites of this study

Dongting Lake (28°30′–30°20′N, 111°40′–113°10′E) is China's second largest freshwater lake with a surface area of 2,625 km^2^. It is the first Yangtze River‐connected lake, located downstream of the Three Gorges Dam with a distance of approximately 300 km (Figure 1). This region has a predominantly subtropical monsoon climate, an annual mean temperature of 16.8°C and a precipitation of 1,382 mm. Dongting Lake is characterized by large seasonal water level fluctuations, which range from up to approximately 36 m in the summer to below 20 m in the winter. The flooding and nonflooding periods generally range from June to September and from October to May of the next year, respectively. Sedge *Carex* meadows, the dominant plant community in Dongting Lake wetland, are mainly distributed along the 23.5–25.4 m elevations. Owing to the periodic inundation pattern of the lake, *Carex* spp. usually have two growing seasons. Typically, they exhibit rapid vegetative growth after flooding (usually from mid‐October to late October) to December; as temperature decreases, the aboveground plant parts turn withered in January. Then, *Carex* spp. of the second growing season sprout again and keep growing until flooding (Deng et al., 2013). The present study was confined to Daxiaoxi Lake, which is located in northwest East Dongting Lake Wetland Reserve (Figure 1). It is one of the most important sublakes within the reserve and also one of the most preferred areas by herbivorous geese with the least amount of human disturbance (Zou et al., 2019).

### 2.2 Hydrological data and remote sensing

#### 2.2.1 Hydrological data

We obtained daily water level data (at 8:00 a.m.) during the two wintering periods (2016/2017 and 2017/2018), from early August to mid‐April in the next year, which covers the entire wintering period of geese, from the Hydrology Inquiry System of Hunan Province.

#### 2.2.2 Normalized difference vegetation index data

The normalized difference vegetation index (NDVI) has been widely used to quantify the food conditions of herbivores (Doiron, Legagneux, Gauthier, & Lévesque, 2013). Based on a previous study in Dongting Lake (Zou et al., 2017), the NDVI of the sedge meadow was used to evaluate the overall growth condition of the meadow in our study area and to reflect the growth status of the traditional main food resource (*Carex* spp.) of the wintering geese. We used the MOD09Q1 dataset provided by the Earth Resources Observation Systems (EROS) data center, the United States Geological Survey (USGS), at a spatial resolution of 250 m and 8‐day interval. The NDVI was calculated with NIR and red band from the MOD09Q1 dataset following the equation of NDVI = (nir − red)/(nir + red). NDVI and hydrological data were collected from the same period.

### 2.3 Field survey

#### 2.3.1 Vegetation survey and laboratory analysis

*Carex* spp. formed monodominant communities in the meadow of our study area. Therefore, we only selected *Carex* spp. for the vegetation survey and further laboratory analysis. In each wintering period, vegetation surveys were conducted in November for the first growing season (this actually extended to early December for the 2017/2018 wintering period owing to the late water recession) and in February for the second growing season. The area from which we collected samples was completely exposed when we started the surveys and was therefore not affected by the subsequent water level fluctuations. For each sampling season, four sampling transects were set up (see Figure 1) and several uniformly spaced quadrats of 1 × 1 m were selected on each transect. Four vegetation variables were measured for each sample: sward height, dry biomass, nitrogen content, and crude fiber content. The former two variables are indicative of the quantity, while the latter two are indicative of the quality of the geese food.

We measured the aboveground height of each quadrat under natural conditions using a steel tape to measure to the nearest 0.1 cm by placing a carton disk on top of the sward. The sward height of a quadrat was determined as the mean of five measurements taken according to a fixed pattern (once from the middle and once from each corner of the quadrat). The aboveground plants were then clipped, stored in plastic bags according to the quadrats they belonged to, and brought back to the laboratory. In the laboratory, the collected samples were re‐examined to remove soil particles, dead material, and other plant species, oven‐dried at 60°C for 48 hr, and weighed for dry mass (g/m^2^). We considered that the abundance of other plant species in the *Carex* meadow was negligible compared with the abundance of *Carex* spp. Therefore, only *Carex* spp. were used for further analysis. A mixed subsample was taken from each sample for the food quality analysis, that is, nitrogen content (% of dry mass) and crude fiber content (% of dry mass). Nitrogen content was measured by an elemental analyzer (Vario MAX CN; Elementar, Langenselbold, Germany), and crude fiber content was measured using a fiber analyzer (Fibretherm FT 12; Gerhardt, Königswinter, Germany). The four vegetation index values were averaged across all the quadrats for each sampling season. Owing to the early development of the *Carex* meadow during its first growing season, our February 2017 survey showed an obvious distinction between the swards developed during the first and the second growing seasons. Therefore, the plants collected in February 2017 were divided into withered and juvenile *Carex* for each quadrat and the aforementioned four variables were measured separately. However, *Carex* swards were generally short and new shoots kept emerging throughout the 2017/2018 wintering period, making it difficult to distinguish between grown‐up and juvenile *Carex*. The sampled plants were thus not classified.

#### 2.3.2 Geese counting

The geese were counted concurrently with the vegetation survey period, that is, from November to early December and during February. Four to five surveys were conducted during each stage during the 8–11 a.m. and the 2–5 p.m. periods. We counted the geese with a spotting scope at the edge of the reserve area to minimize disturbance. The survey spots where we stood to count the geese were mainly distributed along the levee at the edge of the wetland and allowed us to overlook the survey area (see Figure 1). As our aim was to examine the feeding habitat selection of the geese populations foraging on the land area, only foraging geese were counted. Based on the landscape feature of the study area, the habitat used by herbivorous geese was classified into two types: meadow, which consisted mostly of *Carex* spp. meadows, and mudflat, which included bare mudflats, sandbanks, and slightly higher mudflats covered by small sedge, grass, and dicotyledon clones.

### 2.4 Diet analysis

The diet composition of the geese was determined by fecal analysis according to the method suggested by Markkola et al. (2003) and Owen (1975). Three collections of geese droppings were conducted during each sampling season, and approximately 50 fresh droppings from each goose species were collected during each collection. The droppings of Lesser white‐fronted geese were easily discriminated because they were significantly thinner and shorter than those of Bean geese. We neglected the possible existence of droppings from another sympatric herbivorous goose species, the Greater white‐fronted goose, because its population size is very limited compared with those of the Lesser white‐fronted and Bean geese. After we oven‐dried the droppings at 60 ℃ for 24 hr, 30 droppings per species were selected randomly among the samples, mixed thoroughly, and sieved through a 0.15‐mm mesh screen to remove large and thick particles. Then, a subsample of 0.2 g was diluted in 70% ethanol and left overnight before microscopy analysis. Five microscope slides were prepared, and a total of approximately 200 epidermal fragments were photographed for each subsample under a microscope (at ×100 magnification) using a QHY5P‐II camera (Light Speed Vision [Beijing] Co., Ltd). The area of each fragment was estimated using the Digimizer software (version 4.2.6.0).

Samples from plants that were available to the geese were collected concurrently with the droppings from the same area. Epidermis slides for each plant were prepared as reference samples and photographed in the same way as the fecal fragments. The fecal epidermal fragments were identified by comparing them with the photographs of the reference samples. By combining our observations with the results of previous studies on geese diet in Dongting Lake (Cong, Wang, Cao, & Fox, 2012; Guan, Debashish, et al., 2016; Wang, Fox, Cong, & Cao, 2013), the fecal fragments were classified into four classes: (a) *Carex* spp.; (b) monocotyledons (including *Eleocharis*, *Alopecurus*, *Cynodon*, and *Phalaris*); (c) dicotyledons; and (d) others (including nonleaf tissues, unclassified, and a few unidentified ones). Although *Carex* spp. belong to monocots, they were classified separately owing to their abundance in the study area. The proportion of each plant class *i* (*P_i_*) in the diet was calculated as follows (Wang et al., 2013):

$$P_{i}=\frac{A_{i}}{\sum A_{i}},$$

where *A_i_* is the total area of group *i* in the diet.

### 2.5 Clipping control experiment

To examine how the swards that developed during the first growing season affected the feeding patch selection of geese in the second growing season (in February) during the 2016/2017 wintering period, we set up a control experiment in Daxiaoxi Lake. In the *Carex* meadow, three replicated transects near and parallel to water edge were selected. Each replicate was divided into three equally sized quadrats (20 m × 20 m), each of which received a different clipping treatment at the end of the first growing season of 2016/2017. The treatments were as follows: clipped to approximately 5 cm or 20 cm height and no clipping (Figure 5a). The number of droppings was used to denote geese visitation. Before counting the droppings, we removed all the droppings from all the quadrats to ensure the same background values. Then, in February 2017, the droppings of the two goose species were collected and removed from each quadrat twice (in mid‐February and late February) and the numbers were recorded separately. For each transect, we calculated the percentage of geese droppings, which was proportionate to the number of droppings in each quadrat in the total number of geese droppings in one transect.

### 2.6 Statistical analysis

First, we used analysis of variance (ANOVA) to examine whether food quantity and quality differed among the different sampling seasons. We checked the homogeneity of variance before performing ANOVA. If homogeneity of variance held true, one‐way ANOVA was used; otherwise, Welch's ANOVA was used. Both ANOVA types were followed by a post hoc pairwise comparison test (Games–Howell) to investigate the differences.

Then, we considered the two winters as having different water recession patterns (early water recession in 2016/2017 and late water recession in 2017/2018, see detail in Section 3.1). We used “water recession pattern” and “growing season” as fixed factors to test for differences in the overall habitat selection and diet composition of the geese, by applying permutational multivariate analysis of variance (PERMANOVA), which is a nonparametric method to conduct multivariate ANOVA (Anderson & Walsh, 2013). Following the PERMANOVA, which indicated that the geese displayed significantly different habitat selection and diet composition in different winters, we used Mann–Whitney U test (owing to the small sample size for each growing season during each winter) to further examine the differences in each habitat and food type between winters.

Finally, we used ANOVA in the control experiment to examine the effect of sward height on the feeding patch selection of Lesser white‐fronted and Bean geese. Welch's ANOVA was used for Lesser white‐fronted geese because the homogeneity of variance held false. Both ANOVA types were followed by Games–Howell post hoc tests. Independent sample *t* test was used to examine the differences between the two goose species. Data were ln (*x* + 1) transformed for all the analysis.

## 3 RESULTS

### 3.1 Water level and food condition changes

**FIGURE 2:**
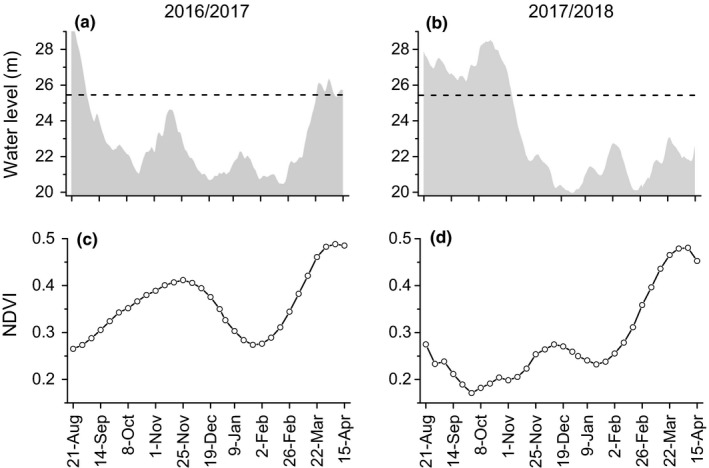
Variations in (a, b) water level and (c, d) NDVI from late August to mid‐April of 2016/2017 and 2017/2018 wintering periods. The dash line in the upper panels denotes water level of 25.4 m, at which the *Carex* spp. meadow are entirely exposed (Zou et al., 2017)

In the wintering periods of 2016/2017 and 2017/2018, water level and NDVI variations were drastically different (Figure 2). In 2016/2017, water level receded in early September, while in 2017/2018 receded in early November. Early water recession in 2016/2017 resulted in much higher NDVI than 2017/2018 during the first growing season. On the other hand, the food quantity and quality differed significantly depending on sampling season (all *p* < .001, see Table 1). The sward height and dry biomass were significantly higher in both growing seasons of 2016/2017 than in those of 2017/2018. The nitrogen content was significantly higher in 2017/2018 with late water recession, while fiber content was significantly lower.

**Table 1:** Comparison of *Carex* spp. quantity (sward height and dry biomass) and quality (nitrogen and crude fiber content) in terms of sampling season (*df* = 3). Plants were divided into withered and new shoots during the second growing season of 2016/2017, but not in 2017/2018 (see detail in Materials and methods)

	Sward height (cm)	Dry biomass (g/m^2^)	Nitrogen content (%)	Crude fiber content (%)	Sample size
Early water recession (2016/2017)					
First growing season	39.00 ± 0.93^a^	362.13 ± 11.71^a^	1.95 ± 0.03^c^	26.81 ± 0.68^a^	38
Second growing season					
Total	36.41 ± 1.22^a^	393.26 ± 16.67^a^	1.72 ± 0.04^d^	28.39 ± 0.62^a^	36
Withered *Carex*	36.41 ± 1.22	314.26 ± 14.57	1.38 ± 0.04	29.45 ± 0.67	
Juvenile *Carex*	15.68 ± 1.49	79.00 ± 8.40	3.05 ± 0.04	24.62 ± 0.58	
Late water recession (2017/2018)					
First growing season	13.55 ± 0.74^c^	52.43 ± 3.80^c^	3.37 ± 0.04^a^	23.33 ± 0.57^b^	42
Second growing season	20.10 ± 1.07^b^	128.94 ± 6.85^b^	2.81 ± 0.09^b^	23.18 ± 0.50^b^	42
*P* value	< 0.001	< 0.001	< 0.001	< 0.001	

Note

Data are expressed as mean ± *SE*. For each vegetation index, different letters indicate significant differences at the .05 significance level.

### 3.2 Habitat preference and diet composition of the two goose species

**FIGURE 3:**
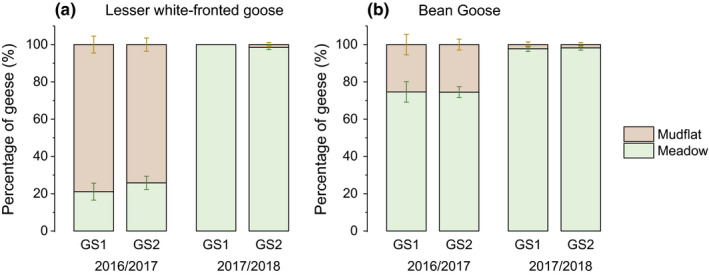
Habitat selection of (a) Lesser white‐fronted geese and (b) Bean geese. GS1 = the first growing season; GS2 = the second growing season

**FIGURE 4:**
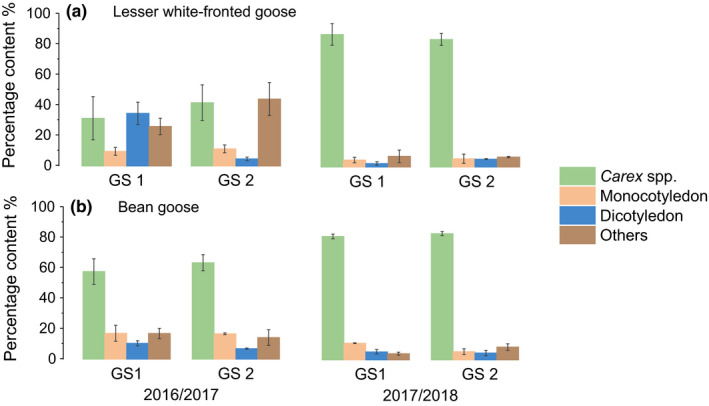
Diet composition of (a) Lesser white‐fronted geese and (b) Bean geese during the study period. GS1 = the first growing season; GS2 = the second growing season

A total of 19 surveys were conducted during the study period (see Table S1). The PERMANOVA results revealed a significant multivariate main effect of the “water recession pattern” (all *p* < .01), but not of the “growing season” (all *p* > .05), on the habitat selection and diet composition of both Lesser white‐fronted and Bean geese (Table 2). The interaction effect was significant on the diet of Lesser white‐fronted geese (*p* < .05), indicating significant changes between two growing seasons within one of the wintering period. The percentages of both goose species feeding on meadow were significantly higher in the 2017/2018 wintering period (*p* < .01, Table 3 and Figure 3). Lesser white‐fronted geese switched from feeding largely on mudflat in 2016/2017 (78.88% ± 4.55%) to feeding entirely on meadow in 2017/2018 (100%), while Bean geese largely utilized the meadow throughout the entire study period (Figure 3). The *Carex* spp. content was significantly higher in both species and dominated their diet in 2017/2018 (Table 3 and Figure 4). “Dicotyledons” and “others” made up an important proportion of the feces of Lesser white‐fronted geese in 2016/2017 with early water recession (Figure 4).

**Table 2:** Summary of permutational multivariate analysis of variance (PERMANOVA, *F* values) for habitat selection and diet composition of Lesser white‐fronted geese and Bean geese in relation to “water recession pattern” (*df* = 1) and “growing season” (*df* = 1) and their interactive effects (*df* = 1)

Source	Water recession pattern (W)	Growing season (G)	W × G
Habitat selection			
Lesser white‐fronted geese	402.15^***^	3.81^NS^	4.33^NS^
Bean geese	15.41^**^	0.08^NS^	0.22^NS^
Diet composition			
Lesser white‐fronted geese	13.85^**^	2.02^NS^	4.81^*^
Bean geese	8.32^**^	0.86^NS^	2.00^NS^

Note

Asterisks denote significant levels (*** *p* < .001; ** *p* < .01; * *p* < .05; NS *p* > .05).

**Table 3:** Mann–Whitney U tests assessing differences in geese habitat selection and diet composition between two wintering periods. Values in the table are the corresponding *p*‐values

Species	Habitat selection			Diet composition				
	Meadow	Mudflat	Sample size	*Carex* spp.	Monocotyledons	Dicotyledons	Others	Sample size
Lesser white‐fronted geese	< 0.001^***^	< 0.001^***^	19	0.016^*^	0.063^NS^	0.286^NS^	0.016^*^	12
Bean geese	0.005^**^	0.005^**^	19	0.004^**^	0.03^*^	0.052^NS^	0.052^NS^	12

Note

Asterisks denote significant levels (*** *p* < .001; ** *p* < .01; * *p* < .05; NS *p* > .05).

### 3.3 The clipping control experiment

**FIGURE 5:**
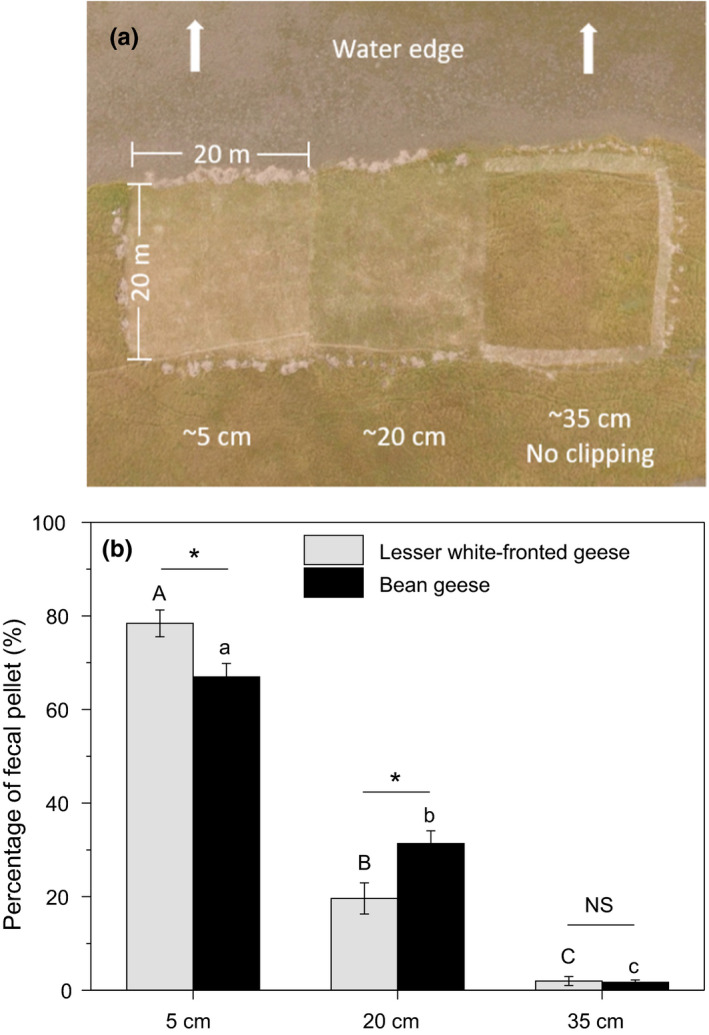
Control experiment testing the effect of sward height on feeding patch selection of the herbivorous geese during the second growing season of 2016/2017. (a) Aerial photograph of one of the experimental transect; (b) percentage of geese droppings in the quadrats with different sward height. Different letters indicate significant differences between treatments at the 0.05 significance level. Asterisks denote significant difference between species (* *p* < .05; NS *p* > 0)

Sward height significantly affected the feeding patch selection of both Lesser white‐fronted geese (*p* < .001) and Bean geese (*p* < .001; Figure 5b), with the largest proportion of geese feeding on the 5 cm patches. In addition, the proportion of Lesser white‐fronted geese that fed on the 5 cm patches was significantly higher than that of Bean geese (*p* < .05); the opposite was true for the 20 cm patches (*p* < .05).

## 4 DISCUSSION

The results of this study indicated that under contrasting water regimes in two successive wintering periods, herbivorous geese selected significantly different habitat types and food items, which may be associated with the major differences in food conditions. A recent study in Poyang Lake, which is another Yangtze River‐connected lake downstream of the Three Gorges Dam, reached a similar conclusion (Aharon‐Rotman et al., 2017). The early water recession in 2016/2017 wintering period reduced the food quality of the abundant *Carex* meadow after geese arrived in Dongting Lake and resulted in the geese, especially the Lesser white‐fronted geese, to trade food quantity for quality; in the winter of 2017/2018, however, almost all the geese fed on meadow and selected *Carex* spp., which could have been an outcome of the overall low plant biomass level and higher food quality. In addition, when facing the same habitat change, the two sympatric goose species exhibited similar but not identical responses, which may be associated with their body sizes. The smaller‐sized Lesser white‐fronted goose, a globally vulnerable species, was more sensitive to habitat change.

In natural lake wetlands in the Yangtze River floodplain, the habitat availability of waterbirds is closely associated with water level fluctuations (Li, Li, et al., 2019). In Dongting Lake, *Carex* spp. shoots emerge immediately after flooding and colonize the exposed substrate rapidly (Chen et al., 2014). In this study, the water recession time in 2016/2017 was around early September, which was approximately two months earlier than in 2017/18 (around early November; Figure 2a,b). This led to a drastic difference between the NDVI of the meadow of the two wintering periods during early to mid‐winter (Figure 2c,d). The results of our field vegetation survey further confirmed the effect of water recession pattern and suggested that the prolonged growing season of the meadow in 2016/2017 that resulted from early water recession increased the quantity of *Carex* spp. (evidenced by the higher sward height and dry biomass) and resulted in significantly lower food quality (evidenced by the lower nitrogen and higher fiber contents) compared with that influenced by late water recession (Table 1). On the other hand, the late water recession time in 2017/2018 may also be indicative of lower temperatures, as temperature generally decreases from autumn to winter (as shown in Figure S1), which could prohibit the germination and development of the plants (Schütz & Rave, 1999), further reducing food quantity. However, we should note that the low NDVI and food quantity in the early winter of 2017/2018 may indicate a smaller habitat area and low ingestion efficiency for the geese (Mueller et al., 2008), which may put their survival at risk and further affect their spring migration and future reproduction (Gates, Caithamer, Moritz, & Tacha, 2001). Moreover, the need to avoid lethal boundary and starvation risk may dominate birds’ habitat and food selection. Indeed, we found that in the early winter of 2017/2018, the geese fed on farmland with intense human activities, which was nearby the protected wetland area; this is something that seldom happens in China (Zhao, Wang, Cao, & Fox, 2018).

High food quantity and low food quality are generally unfavorable to herbivores (Hassall, Riddington, & Helden, 2001). Increasing the sward height to a certain level could increase the peck size of grazing Anatidae; however, this could also increase the cropping time, especially in smaller species, which could lead to a significant decline in intake rate (Durant et al., 2003). Patch selection was therefore affected. In 2016/2017 wintering period, the tall swards and large biomass of the *Carex* meadow may have led to an increased searching and handling effort in the smaller Lesser white‐fronted geese and resulted in a type IV functional response (Heuermann, Langevelde, Van Wieren, & Prins, 2011). Therefore, the geese switched to feed on the later exposed mudflat. On the other hand, the nitrogen content of *Carex* spp. in 2016/2017 could have been indicative of low nitrogen ingestion and result in negative nitrogen budgets in herbivorous geese (Wang et al., 2014); the concurrent significantly higher crude fiber content may indicate low food digestibility. All these factors could influence the ability of the geese to satisfy their energy and nutrient requirements. In the food quantity–quality trade‐off, the larger body size benefited Bean geese, which normally follow a type II functional response (Zhang et al., 2016), to exploit the taller sward and lower quality food in the 2016/2017 wintering period. As a result, more Bean geese fed on meadow than Lesser white‐fronted geese (Figure 3). The contrasting food conditions led to significant changes in the habitat preferences of both goose species from 2016/2017 to 2017/2018 (Table 2). Nearly all the observed populations selected to feed on meadow in both growing seasons of 2017/2018. Correspondingly, *Carex* spp. dominated the geese diet in 2017/2018 (Figure 4 and Table 3).

Previous studies showed that dicotyledons constitute only a small proportion of the diet of Lesser white‐fronted geese in both of their breeding (Markkola et al., 2003; Rozenfeld & Sheremetyev, 2016) and wintering sites (Cong et al., 2012; Karmiris, Kazantzidis, Platis, & Papachristou, 2017; Wang et al., 2013). In the present study, however, dicotyledons accounted for approximately a third of the Lesser white‐fronted geese’ diet in the early winter of 2016 (Figure 4a). Furthermore, according to visual observations and later in situ confirmation, we detected Lesser white‐fronted geese feeding on mosses and grub for below‐ground plant parts on mudflat. Reflected in our results was the relatively high proportion of “others” in the geese's diet during 2016/2017. However, the proportion of moss in geese diet may be underestimated as moss leaves consist mainly of monolayer cells (Wu et al., 1982), which may make them easily digestible by the geese. Although moss is used by several small goose species such as the Brent goose (*Branta bernicla*) and Barnacle goose (*Branta leucopsis*) (Madsen, Bregnballe, & Mehlum, 1989; Soininen, Hubner, & Jonsdottir, 2010), to our knowledge, there have been no reports on the exploitation of moss by Lesser white‐fronted geese. We considered the dietary choice of this species in 2016/17 wintering period to be a forced strategy under severe food stress caused by extremely early water recession. Lei et al. (2019) used stable isotope analysis and also showed the effect of poor habitat conditions on both goose species in the wintering period of 2016/2017 in East Dongting Lake; they found that both species had different diets compared with the previous winter when the occurrence of the water recession was in an appropriate time (in mid‐October).

Food availability is a composite variable depending on both the abundance and accessibility of food (Lantz, Gawlik, & Cook, 2015). Although the newly generated shoots in the second growing season of 2016/2017 were suitable food for the geese (Table 1), the effect of “growing season” was not significant on either goose species. We ascribe this to the negative influence of the excessively tall swards that developed in the first growing season, which greatly decreased the accessibility to the newly generated, nutritious food and kept the geese away from the sedge meadow. The result of the control experiment, in which both of the goose species fed largely on the clipped patches (Figure 5), supported our inference. Removing the tall, withered plants facilitated the geese to feed on the newly generated swards. Both of the goose species preferred the clipped patches with the 5‐cm‐tall swards the most. This result also proved that grazing animals can perceive differences among feeding stations and small patches (Bailey et al., 1996).

Meadow was the major feeding habitat of Bean geese, while *Carex* spp. dominated their diet throughout the two wintering periods. The shifts in habitat and food selection in Bean geese from 2016/2017 to 2017/2018 were not as notable as in Lesser white‐fronted geese. Moreover, the clipping experiment showed that the proportion of Bean geese feeding on the 20 cm patches was significantly higher than that of Lesser white‐fronted geese. These results suggest that Bean geese were more capable of utilizing taller swards and feeding in patches with taller swards (for shorter plants) and may be less susceptible to quality food shortages than Lesser white‐fronted geese (Zhang et al., 2018). We inferred that the morphological and physiological characteristics of the two goose species could account for their different habitat use and diet selection patterns. First, the grazing intake rates varied with geese body and bill morphology. The relatively short neck and beak of Lesser white‐fronted geese limit their utilization of plants with taller swards and higher biomass. Secondly, size‐related digestive capabilities may make the smaller Lesser white‐fronted goose less capable of utilizing poor quality plants (Van Soest, 1996). Finally, we inferred that, in East Dongting Lake, the gut microbiota of Lesser white‐fronted geese were not as diverse and functional in food digestion and absorption as they were in Bean geese. This assumption was based on a research carried out in Yangtze River wetlands, which suggested that the characteristics of the microbial community structure of Bean geese feces may make them more beneficial in digesting a nutritionally poor diet and may facilitate cellulose degradation and nutrient absorption (Yang, Deng, & Cao, 2016). Our inference, however, requires further confirmation. In addition, interspecific competition is likely to also influence and shape strategy choice of animals. This could be an interesting next step to improve our understanding of the response of herbivorous geese to habitat change in East Dongting Lake wetland.

## 5 CONSERVATION IMPLICATIONS

East Dongting Lake is a Ramsar site that supports globally important waterbird populations, especially the globally vulnerable Lesser white‐fronted goose. Therefore, its habitat quality does not only have local, but also global significance for habitat and biodiversity conservation. Dam constructions have significant impacts on downstream lakes (Nilsson, Reidy, Dynesius, & Revenga, 2005). As the first Yangtze River‐connected lake located downstream of the Three Gorges Dam, the hydrological regime and habitat quality of Dongting Lake are obviously affected by dam operation (Sun, Huang, Opp, Hennig, & Marold, 2012; Xie et al., 2015). As the Administration Bureau of East Dongting Lake National Nature Reserve cannot manage the Three Gorges Dam directly, it would be possible to adopt conservation and management approaches within the East Dongting Lake wetland. Such approaches could include water control in the sublakes to better manage the sedge meadow growth, thereby improving the ingestion and digestibility of the vegetation by the geese or additional food supplies (e.g., planting winter wheat) for the geese to make up for the quality and quantity deficiencies. Predictions on timing of water recession could be made by combining historical and current hydrological data to better regulate the water levels. Furthermore, human disturbances are significantly associated with waterbird abundance in the Yangtze River wetlands (Yuan et al., 2014; Zhang et al., 2019). In Dongting Lake, human disturbances mainly include fishing activities, reed harvesting, and recreational activities (Zou et al., 2019). Therefore, enhanced supervision measures need to be adopted in order to minimize human disturbances in the reserve, especially during wintering periods when quality food resources are lacking.

## CONFLICT OF INTEREST

The authors declare that there is no conflict of interest.

## AUTHOR CONTRIBUTIONS

**Pingyang Zhang:** Conceptualization (lead); Formal analysis (supporting); Investigation (lead); Methodology (equal); Validation (lead); Writing‐original draft (lead); Writing‐review & editing (lead). **Ye‐ai Zou:** Conceptualization (lead); Formal analysis (equal); Funding acquisition (lead); Methodology (equal); Project administration (lead); Supervision (supporting); Writing‐original draft (eqal); Writing‐review & editing (equal). **Yonghong Xie:** Conceptualization (lead); Funding acquisition (supporting); Supervision (lead); Writing‐original draft (eqal); Writing‐review & editing (equal). **Siqi Zhang:** Conceptualization (supporting); Formal analysis (supporting); Investigation (equal); Methodology (equal); Writing‐review & editing (supporting). **Xinsheng Chen:** Conceptualization (supporting); Formal analysis (equal); Funding acquisition (supporting); Methodology (equal); Writing‐original draft (supporting); Writing‐review & editing (supporting). **Feng Li:** Conceptualization (supporting); Formal analysis (equal); Funding acquisition (supporting); Methodology (supporting); Writing‐original draft (supporting). **Zhengmiao Deng:** Conceptualization (supporting); Formal analysis (equal); Funding acquisition (supporting); Methodology (equal); Writing‐original draft (supporting). **Hong Zhang:** Investigation (lead); Project administration (equal); Resources (equal); Writing‐original draft (supporting). **Wei Tu:** Investigation (equal); Methodology (equal); Project administration (supporting); Resources (supporting).

P.Z., Y.Z., and Y.X.: Conceptualization of idea and study design. P.Z., S.Z., H.Z., and W.T. Conduct the experiment and sample analysis. Y.Z., X.C., F.L., and Z.D: Data collection and conduct the data analysis. P.Z. Manuscript writing with the help of all the other authors. All authors read and approved the final manuscript.

## Supporting information

Figure S1

Table S1

## Data Availability

Relevant data will be available via Dryad: https://doi.org/10.5061/dryad.8w9ghx3hc.
