# Supplementary figures and images for: Hydrology‐driven responses of herbivorous geese in relation to changes in food quantity and quality
Source: Ecol Evol. 2020 Apr 20;10(12):5281–92. doi: 10.1002/ece3.6272 (PMC7319142; doi:10.1002/ece3.6272)

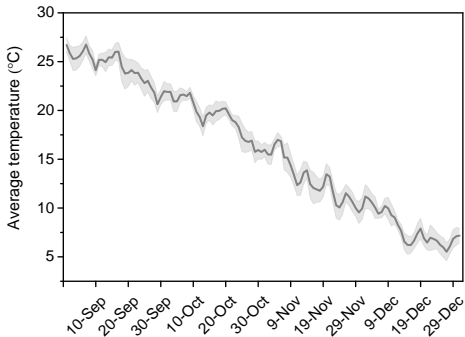

Supplement: Supplementary file 1 — Figure S1 [file ECE3-10-5281-s001.pdf]
