# Supplementary material for: Hydrology‐driven responses of herbivorous geese in relation to changes in food quantity and quality
Source: Ecol Evol. 2020 Apr 20;10(12):5281–92. doi: 10.1002/ece3.6272 (PMC7319142; doi:10.1002/ece3.6272)
Supplement: Supplementary file 2 — Table S1 [file ECE3-10-5281-s002.doc]

# Appendix

TABLE A1 Summary of the habitat selection of Lesser white-fronted geese and Bean geese in different stages of the 2016/2017 and 2017/2018 wintering periods. GS1 = the first growing season; GS2 = the second growing season

|  | Lesser white-fronted geese | | | | Bean geese | | | |
| --- | --- | --- | --- | --- | --- | --- | --- | --- |
|  | Number of feeding geese | | Percentage of feeding geese | | Number of feeding geese | | Percentage of feeding geese | |
| Stage | Meadow (ind.) | Mudflat (ind.) | Meadow  (%) | Mudflat  (%) | Meadow (ind.) | Mudflat (ind.) | Meadow  (%) | Mudflat  (%) |
| 2016/2017 GS1 | 30 | 155 | 16.22 | 83.78 | 197 | 53 | 78.80 | 21.20 |
|  | 39 | 109 | 26.35 | 73.65 | 86 | 10 | 89.58 | 10.42 |
|  | 18 | 60 | 23.08 | 76.92 | 200 | 160 | 55.56 | 44.44 |
|  | 20 | 40 | 33.33 | 66.67 | 140 | 48 | 74.47 | 25.53 |
|  | 17 | 240 | 6.61 | 93.39 | 47 | 16 | 74.60 | 25.40 |
| 2016/2017 GS2 | 41 | 184 | 18.22 | 81.78 | 137 | 58 | 70.26 | 29.74 |
|  | 180 | 580 | 23.68 | 76.32 | 20 | 9 | 68.97 | 31.03 |
|  | 108 | 198 | 35.29 | 64.71 | 185 | 53 | 77.73 | 22.27 |
|  | 100 | 282 | 26.18 | 73.82 | 86 | 20 | 81.13 | 18.87 |
| 2017/2018 GS1 | 10 | 0 | 100.00 | 0.00 | 607 | 22 | 96.50 | 3.50 |
|  | 15 | 0 | 100.00 | 0.00 | 490 | 2 | 99.59 | 0.41 |
|  | 60 | 0 | 100.00 | 0.00 | 316 | 0 | 100.00 | 0.00 |
|  | 25 | 0 | 100.00 | 0.00 | 304 | 24 | 92.68 | 7.32 |
|  | 18 | 0 | 100.00 | 0.00 | 79 | 0 | 100.00 | 0.00 |
| 2017/2018 GS2 | 248 | 0 | 100.00 | 0.00 | 60 | 0 | 100.00 | 0.00 |
|  | 269 | 0 | 100.00 | 0.00 | 401 | 0 | 100.00 | 0.00 |
|  | 61 | 4 | 93.85 | 6.15 | 666 | 43 | 93.94 | 6.06 |
|  | 127 | 0 | 100.00 | 0.00 | 54 | 0 | 100.00 | 0.00 |
|  | 379 | 5 | 98.70 | 1.30 | 481 | 13 | 97.37 | 2.63 |

FIGURE A1 Daily temperature variations in Dongting Lake area from early September to late December. Dark grey line indicates multi-year average (2007-2017) and light grey area indicates standard error
